# A Comprehensive Review of the Role of Biologics and Small-Molecule Therapies in the Long-Term Management of Crohn’s Disease

**DOI:** 10.7759/cureus.92901

**Published:** 2025-09-22

**Authors:** Syeda F Zaidi, Keval B Patel, Maria Gabriela Cerdas, Ekanta Timalsina, Abirami Rajendiran, Duaa Behbehani, Sara Mahmood Aljallawi, Gnaneswar Pendurthi, Saad Ullah, Mounia N Bellaha, Tsion Gebregiorgis, Zahra Nazir

**Affiliations:** 1 Internal Medicine, University College of Medicine and Dentistry, Lahore, PAK; 2 Surgery, Narendra Modi Medical College, Ahmedabad, IND; 3 Medicine, Universidad de Ciencias Médicas, San Jose, CRI; 4 General Practice, Chitwan Medical College, Bharatpur, NPL; 5 Internal Medicine, Texas Tech University Health Sciences Center El Paso, Paul L. Foster School of Medicine, El Paso, USA; 6 Internal Medicine, East Lancashire Hospitals National Health Service (NHS) Trust, Blackburn, GBR; 7 General Practice, Mansoura University, Mansoura, EGY; 8 General Medicine, Guntur Medical College, Guntur, IND; 9 Medicine and Surgery, University of Health Sciences Lahore, Lahore, PAK; 10 Veterinary Medicine, American Veterinary Medical Association (AVMA), Schaumburg, USA; 11 General Practice, Faculty of Medicine, Addis Ababa University, Addis Ababa, ETH; 12 Internal Medicine, Combined Military Hospitals, Quetta, PAK

**Keywords:** biologic treatment, crohn’s disease, janus kinase inhibitor, small-molecule agents, ulcerative colitis (uc)

## Abstract

Crohn’s disease (CD), a chronic inflammatory condition of the gastrointestinal tract, poses significant therapeutic challenges due to its relapsing course and complex pathophysiology. With the rapid expansion of biologic agents and small-molecule therapies, clinicians face increasing complexity in selecting and sequencing treatment options. To address this, we conducted an extensive literature review across various databases, including PubMed and Google Scholar, focusing on studies published within the last decade. Inclusion criteria encompassed randomized controlled trials, cohort studies, and narrative reviews that evaluated adult and middle-aged populations. Case reports, non-peer-reviewed sources, and studies with limited follow-up data were excluded from the analysis.

This review examines the mechanisms of action, clinical efficacy, safety profiles, and treatment positioning of biologic agents, such as anti-TNF therapies, anti-integrins, and IL-12/23 inhibitors, as well as small-molecule therapies, including JAK inhibitors and S1P receptor modulators. We also explore emerging strategies such as early biologic initiation, combination regimens, and personalized approaches to therapy. Upon evaluating statistical data from the included studies, we conclude that biologics and small-molecule therapies currently constitute the cornerstone of long-term management in moderate to severe CD. This review aims to synthesize contemporary clinical evidence and provide insights into future therapeutic directions.

## Introduction and background

Crohn’s disease (CD) is one of the two majorly known inflammatory bowel diseases (IBDs) of the gastrointestinal tract that presents as discontinuous, patchy, and transmural inflammation. This process involves the entire span of the gut but is more commonly seen in the terminal ileum. This disease has periods of flare and remission and often presents with extra-intestinal manifestations. The etiology of CD is multifactorial, resulting from the interaction of environmental factors, dysregulation of the innate immune system, genetic susceptibility, and gut dysbiosis [1].

According to the study by Lewis et al., the age- and sex-standardized incidence of IBD per 100,000 person-years in the United States was 10.9 (95% CI, 10.6-11.2). The incidence of IBD (ulcerative colitis (UC) and CD) peaked in the third decade of life, decreased to a relatively stable level across the fourth to eighth decades, and declined further beyond the age of 80. The study reported an overall higher incidence of UC in adults (6.3; 95% CI, 6.1-6.6) compared to CD (4.1; 95% CI, 3.9-4.3). Data showed children have a higher incidence of CD compared to UC. This study also estimated that relative to Black individuals, Asian individuals, and Hispanic American individuals, there are seven, 21, and six times more White American individuals with IBD [2].

**Figure 1:**
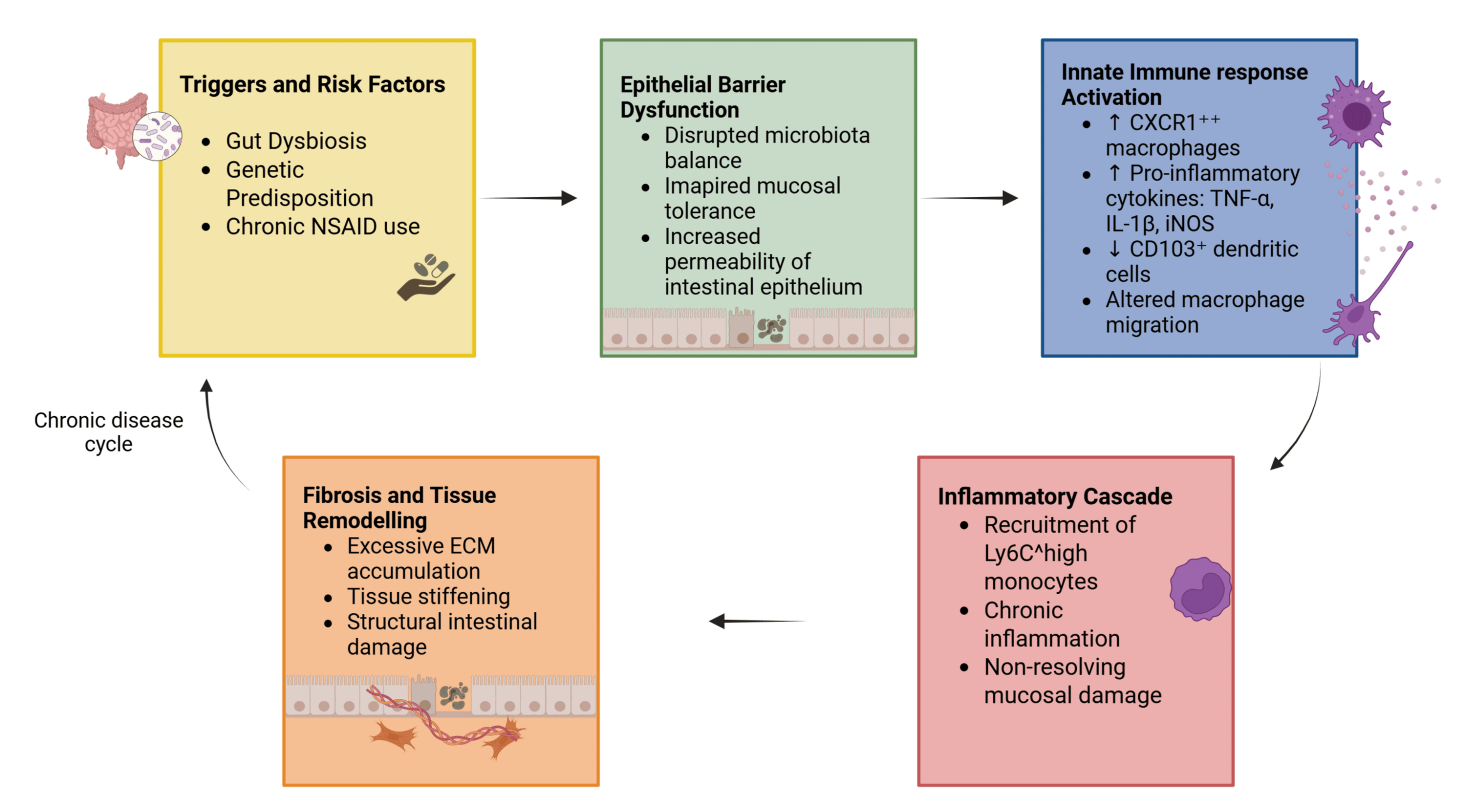
Pathophysiology of CD CD: Crohn’s disease, NSAID: non-steroidal anti-inflammatory drug, ECM: extracellular matrix, CXCR1: C-X-C chemokine receptor type 1, TNF-α: tumor necrosis factor-alpha, IL-1β: interleukin-1 beta, iNOS: inducible nitric oxide synthase, CD103: cluster of differentiation 103, Ly6C: lymphocyte antigen 6 complex, locus C Source reference: [3]. Created using biorender.com.

IBD is sustained in genetically susceptible individuals by an impaired immune response against intestinal microorganisms and is associated with the dysregulation of both innate and adaptive immune responses. The disease presents with infiltration of the intestinal epithelial barrier by immune cells, leading to chronic inflammation and non-resolving mucosal damage, which is considered a hallmark characteristic of the disease [3]. While the exact cause remains unknown, potential triggers include infectious agents, chemical compounds (non-steroidal anti-inflammatory drugs), and metabolic alterations potentially linked to diet-mediated dysbiosis. Dysbiosis disrupts the balance of the intestinal microbiota, impairing tolerance mechanisms and enabling the activation of CXCR1high macrophages and other immune cells, which secrete pro-inflammatory cytokines such as tumor necrosis factor (TNF)-𝛼, interleukin (IL)-β, and inducible nitric oxide synthase. This leads to the recruitment of Ly6Chigh inflammatory monocytes, excessive extracellular matrix deposition, and fibrosis, further exacerbating intestinal inflammation. Concurrently, the reduction in CD103+ dendritic cells and changes in macrophage migration patterns contribute to impaired immune regulation, fueling the chronic inflammation characteristic of IBD [3]. The pathophysiology has been depicted in Figure 1.

CD remains a challenging condition to treat. Still, recent advances have provided alternative routes for control and remission, particularly biologics and small-molecule therapies, that offer patients better therapeutic and long-term management options. However, despite their effectiveness, these treatments have associated risks and complications that require careful monitoring. This review aims to compile and analyze existing literature on small-molecule and biologic therapies for CD. By consolidating these findings, this review aims to provide a comprehensive understanding of how these therapies can be optimized to serve future clinical approaches and patient outcomes.

## Review

Managing CD involves a collaborative approach between multidisciplinary teams and the patient. Current therapies for CD include dietary modification, risk factor management, and medical and surgical treatments.

Diet plays a supportive role in the treatment of CD. Input from a dietitian and nutritional supplementation is recommended, as patients with symptoms are at risk for micronutrient deficiencies and malnutrition. For instance, patients with a history of terminal ileitis or ileal resection are more likely to experience vitamin B12 deficiency. Data from pediatric studies suggest that specific dietary approaches, such as elemental and semi-elemental diets, can promote the resolution of mucosal inflammation. Even so, these diets have not been as successful in adults due to poor compliance, and the benefits are often not long-lasting, as inflammation tends to recur once a less restrictive diet is reintroduced [4].

CD is generally split into two phases: induction and maintenance. In the induction phase, treatment aims to control inflammation within three months. The maintenance phase focuses on keeping the patient symptom-free for as long as possible. The choice of treatment depends upon careful assessment of the patient's risk profile and the severity of the disease. For mild-to-moderate cases, commonly used therapies include steroids, methotrexate, oral mesalamine, and immunomodulators such as thiopurines (6-mercaptopurine, azathioprine).

Corticosteroids are primarily used to induce remission during CD flare-ups and to stabilize the condition until immunomodulators or biologic therapies become effective, especially in cases that are moderate to severe. While corticosteroids can be beneficial in the short term, their use as long-term maintenance treatment is discouraged because of the potential for chronic side effects, including osteoporosis, osteonecrosis, and adrenal insufficiency [5]. On the other hand, mesalamines (5-ASA) and sulfasalazine (S-ASA, which contains a sulfapyridine component) possess anti-inflammatory properties, making them effective in UC; however, their benefit in CD needs further study. These agents may be considered for mild colonic CD but are not effective for small bowel involvement [6]. Immunomodulators are steroid-sparing agents used to maintain remission in moderate CD. As they typically take eight to 12 weeks to exert clinical effects, they are not suitable for inducing remission in active disease. However, they may be used in conjunction with corticosteroids during the initial phase of treatment. Effective dosages include azathioprine (1.5-2.5 mg/kg/day), 6-mercaptopurine (0.75-1.5 mg/kg/day), and methotrexate (15-25 mg weekly) [7,8].

Surgical interventions, such as bowel resection, stricturoplasty, or abscess drainage, are required in up to two-thirds of patients with CD during their lifetime. Surgical intervention is indicated in patients who do not respond to medical therapy and, as such, in late-stage disease presenting with perforation, persistent or recurrent bowel obstruction, bowel abscesses that cannot be treated with percutaneous drainage, uncontrolled bleeding, bowel dysplasia, or cancer [8]. The primary reason for surgical resection of the intestine in CD is small bowel obstruction caused by a fibrostenotic stricture. The second most common reason for resection is related to penetrating CD, such as internal fistulas or sinus tracts that lead to abscesses or phlegmon. While intestinal resection is the most effective treatment for strictures, stricturoplasty can be considered a bowel-preserving alternative, especially for patients at risk of short gut syndrome. Managing CD requires a collaborative approach between gastroenterologists and surgeons. Early surgical consultation is recommended for patients with strictures or penetrating complications [9].

Biologic therapies in Crohn's disease

Biologic therapies have been proven to revolutionize the management of CD by targeting specific immune pathways involved in the disease's pathogenesis. These biologic agents can be divided into TNF inhibitors, integrin receptor antagonists, and IL-12/23 inhibitors (Table 1) [10].

**Table 1:** Biologic therapies in CD CD: Crohn’s disease, UC: ulcerative colitis, TNF: tumor necrosis factor, IL: interleukin, RCT: randomized controlled trial Source reference: [4,6-23]

Drug category	Drug name	Mechanism of action	Use and dosage	Clinical efficacy
Anti-TNF agents	Infliximab, adalimumab, certolizumab pegol	Bind to TNF-α, preventing receptor interaction → inhibit downstream pro-inflammatory signaling	Induction and maintenance. Infliximab: IV 5 mg/kg at 0, 2, 6 wks → q8w (ACCENT I, II). Adalimumab: 160 mg SC wk0, 80 mg wk2 → 40 mg q2w (CLASSIC I, CHARM). Certolizumab: 400 mg SC at 0, 2, 4 wks → q4w (PRECiSE 1, 2)	Infliximab: ACCENT I, remission 39% at wk30 vs. 21% placebo; ACCENT II, 36% fistula closure at wk54 vs. 19% placebo. Adalimumab: CLASSIC I, remission 36% at wk4 vs. 12% placebo; CHARM, remission 40% at wk26 vs. 17% placebo. Certolizumab: PRECiSE 1, induction not significant; PRECiSE 2, remission 37% at wk26 vs. 27% placebo (p<0.05)
Anti-IL-12/23 agents	Ustekinumab	Blocks p40 subunit of IL-12/23 → inhibits Th1/Th17 activation	Induction and maintenance. IV induction ~6 mg/kg → SC 90 mg q8w (UNITI-1, UNITI-2, IM-UNITI)	UNITI-1 (anti-TNF failures): remission wk6 = 34.3% vs. 21.5% placebo. UNITI-2 (anti-TNF naïve): remission wk6 = 55.5% vs. 28.7% placebo. IM-UNITI: remission 53.1% at wk44 vs. 35.9% placebo
Anti-IL-23 (p19) agents	Risankizumab, guselkumab, brazikumab, mirikizumab	Block p19 subunit of IL-23 → suppress IL-23–mediated inflammation	Induction and maintenance. Risankizumab: IV 600 mg at 0, 4, 8 wks → SC 180 mg q8w (ADVANCE, FORTIFY). Guselkumab: SC dosing under trial (GALAXI-1). Brazikumab/mirikizumab: phase II/III trials.	Risankizumab: remission 45–55% wk52 (ADVANCE, FORTIFY). Guselkumab: higher remission rates vs. placebo (GALAXI-1). Mirikizumab: endoscopic response 25–44% wk12, 58% wk52
Anti-integrin agents	Vedolizumab, natalizumab	Block integrins → prevent leukocyte trafficking to the gut	Induction and maintenance. Vedolizumab: IV 300 mg at 0, 2, 6 wks → q8w (GEMINI II). Natalizumab: IV 300 mg q4w (ENACT-1, ENCORE)	Vedolizumab: GEMINI II, remission 15% vs. 7% placebo at wk6; maintenance remission 39% vs. 22%. Natalizumab: ENCORE, remission 48% vs. 32% placebo at wk12
Anti-adhesion molecule	Etrolizumab	Targets β7 integrin (α4β7 and αEβ7) → blocks lymphocyte homing	Trial phase only. Tested in CD/UC RCTs, mixed efficacy	Early results: no clear superiority vs. placebo in CD; development ongoing

Mechanism of Action

Anti-TNF agents: Anti-TNF agents such as infliximab, adalimumab, golimumab, and certolizumab pegol target and neutralize TNF-α, a pro-inflammatory cytokine. By blocking TNF-α, these therapies reduce inflammation and promote mucosal healing [11].

Integrin receptor antagonists, such as anti-integrins like vedolizumab, inhibit the α4β7 integrin, preventing leukocyte migration to the gastrointestinal tract. With minimal systemic immunosuppression, this gut-specific mechanism effectively controls intestinal inflammation [12].

IL-12/23 inhibitors: IL inhibitors, including ustekinumab, target the p40 subunit shared by IL-12 and IL-23, cytokines involved in the differentiation and activation of T-cells. By inhibiting these ILs, ustekinumab helps modulate the inflammatory response in CD [11].

Clinical Indication, Efficacy, and Outcomes

Biologics have demonstrated efficacy in inducing and maintaining remission in CD. They are typically indicated for patients with moderate to severe CD who have not responded adequately to conventional treatments such as corticosteroids and immunomodulators.

Anti-TNF agents: Infliximab and adalimumab are effective in achieving and sustaining clinical response and remission in both adults and children with moderate-to-severe CD who did not adequately respond to conventional treatments. In addition, infliximab has been shown to promote mucosal healing, decrease the need for corticosteroids, and efficiently maintain treatment for fistulizing CD [13]. Response rates for infliximab were as high as 90%, with one-year remission rates of 55-60% for luminal CD and a 75% response with 50% remission rates for perianal CD. Adalimumab has shown a 45% remission rate in anti-TNF-naïve children and 20% in those who do not respond to infliximab [14]; later studies have found that combining infliximab with azathioprine is more effective than using either treatment alone in newly diagnosed CD patients, due to thiopurines reducing anti-infliximab antibodies. Additionally, these studies demonstrated that infliximab was the most effective drug at healing ulcers in ileal CD, followed by adalimumab [15].

A review article by Dunleavy et al. demonstrated that anti-TNF therapies achieved higher clinical remission rates than placebo, with infliximab yielding the most favorable results. Furthermore, fistula healing was more effective with infliximab and adalimumab, while certolizumab pegol was less effective. Long-term outcomes indicated that anti-TNF therapies reduced hospitalization and surgery rates, particularly infliximab and adalimumab, while certolizumab pegol showed higher hospitalization risks [16]. Certolizumab pegol has mixed efficacy in CD, with inconsistent results across trials. Golimumab appears effective in refractory cases but needs further examination [11].

Integrin receptor antagonists: Vedolizumab, which inhibits α4β7 integrins to block lymphocyte migration into the gut, is effective in maintaining remission in moderate-to-severe CD. The GEMINI 2 study demonstrated vedolizumab's efficacy in CD, achieving remission in 14.5% of patients by week 6 (vs. 6.8% with placebo) and sustaining remission over one year in 39% and 36.4% of patients on eight-week and four-week regimens, respectively (vs. 21.6% with placebo) [12].

IL-12/23 inhibitors: Ustekinumab, an IL-12/23 inhibitor, effectively induces and maintains remission in moderate-to-severe CD, particularly in patients nonresponsive to anti-TNF therapies. Ustekinumab is also beneficial for treating perianal disease and fistula healing [11]. It has a favorable safety profile and low tuberculosis reactivity and may eventually become a first-line biologic, especially in patients with psoriasis [12].

Safety and Adverse Effects

Biologic agents effectively treat immune-mediated diseases but carry risks like cancer, infections, and tuberculosis reactivation. Despite these risks, patients often prefer biologics over immunomodulators due to better effectiveness and tolerability [17].

TNF-α inhibitors have significant safety concerns, including serious infections, cancers, heart failure, blood disorders, and neurological issues. Live vaccines are not recommended for patients with TNFα inhibitors due to the increased infection risk [13]. Before initiating anti-TNF therapy, patients should undergo screening for tuberculosis and hepatitis B to prevent reactivation, and varicella immunity should be assessed [14]. Recent research shows that IBD patients face increased cardiovascular risks, but therapies like anti-TNF agents may help reduce this risk. Anti-TNFs lower cardiovascular markers but are contraindicated in severe heart failure due to potential mortality risks. Additionally, these studies have shown that the weight gain associated with this medication is more closely related to reduced inflammation than to the drug itself [18].

Newer biologics, such as vedolizumab and ustekinumab, have demonstrated safer profiles with reduced risk of infection or malignancy. Natalizumab is linked to progressive multifocal leukoencephalopathy and should only be used in patients who are negative for anti-John Cunningham virus antibodies. On the other hand, vedolizumab does not have this risk and is preferred due to its targeted action on leukocyte trafficking in the gut. It has been proven effective in achieving clinical response, remission, and sustained remission without corticosteroids [16]. However, recent studies have shown that due to vedolizumab's antagonistic effect on MAdCAM-1, which is also present in the oropharynx, it is believed that it may disrupt a protective mechanism in the upper respiratory mucosa, increasing the risk of respiratory infections [18]. Additionally, the gut-specific mechanism of action of vedolizumab does not elevate the risk of enteric infections. However, it may diminish immune responses to oral cholera vaccines, suggesting a partial suppression of gut-specific T-cells [13].

Ustekinumab's safety profile is comparable to that of other biologics. Common adverse effects include nasopharyngitis, headache, and injection site reactions. The risk of infections is also present, though it appears to be lower than with anti-TNF agents [19].

Emerging Therapies

Dual biologic therapy (DBT) is a strategy developed to target multiple inflammatory pathways to enhance refractory CD outcomes. Vedolizumab and ustekinumab are preferred options due to their safety profiles. A study by Yang et al. investigated DBT for high-risk refractory CD, showing promising results in patients with significant prior treatment failures. DBT led to a 43% improvement in endoscopic findings, clinical remission, and reduced biomarkers, with an adverse event rate of 13% [19].

Guselkumab, a monoclonal antibody targeting IL-23, is currently approved for psoriasis and is being evaluated for CD in the GALAXI-1 (Guselkumab for the Treatment of Crohn's Disease) phase II trial. Preliminary results show that guselkumab is more effective than placebo in achieving clinical remission, with higher remission rates seen in patients with previous biologic failures. Additionally, guselkumab treatment resulted in high clinical and endoscopic efficacy at week 48. Its safety profile is comparable to a placebo, with no significant infection risks. However, phase II/III trials are ongoing [16].

Additionally, a study by Danese et al. assessed the tolerability and efficacy of guselkumab in patients with active CD of moderate to severe intensity. At week 48, guselkumab treatment resulted in high clinical remission rates (57-73%) and endoscopic response rates (44-46%), outperforming both placebo and ustekinumab. These results indicated that guselkumab provided sustained clinical and endoscopic improvements with no new safety issues [20].

Risankizumab and mirikizumab, both targeting IL-23, showed positive clinical and endoscopic improvement results in trials. However, further evaluation in phase IV and comparative studies is needed [11]. A study by Ferrante et al. evaluated subcutaneous risankizumab as a maintenance therapy for CD and found that patients receiving 360 mg of risankizumab had higher rates of clinical remission (52% vs. 41%) and endoscopic response (47% vs. 22%) compared to placebo. These findings demonstrated that subcutaneous risankizumab is well-tolerated and effective for remission in moderate to severe CD [21]. Another study by D'Haens et al. assessed the safety and efficacy of risankizumab for inducing remission in patients with moderate to severe CD. By week 12, risankizumab demonstrated significantly higher clinical remission and endoscopic response rates than placebo. The treatment was well tolerated, with adverse events comparable across groups. These results highlight risankizumab as a promising induction therapy for CD [22].

Similarly, a study by Sands et al. assessed the efficacy of mirikizumab in CD, revealing significantly better endoscopic response rates (25.8-43.8%) at week 12 compared to placebo (10.9%). By week 52, the treatment demonstrated lasting effectiveness, with endoscopic response rates of 58.5-58.7%. The safety profile was similar to that of the placebo, making mirikizumab a promising treatment for CD [23].

Small-molecule therapies

For IBDs, small-molecule medicines (SMDs) have been developed that employ a variety of unique pharmacological routes, such as phosphodiesterase-4 (PDE4) inhibitors, sphingosine-1-phosphate (S1P) receptor modulators, and inhibitors of Janus kinases (JAKs) [24]. SMDs lack a stable structure and have a low molecular weight (<1 kDa) [24]. Typically, they are chemically incompatible with biomolecular medications. This structural variation subsequently impacts their pharmacokinetics, primarily because of a greater diffusion capacity. In fact, due to their small size, SMDs readily diffuse across cell membranes, while bigger molecules can only be found in the extracellular or vascular compartment [24]. Monoclonal antibodies are administered slowly and are typically given monthly or bimonthly. However, their relatively short half-life necessitates frequent oral dosing, often once or twice daily. Their rapid onset of action helps to compensate for this limited duration (Table 2) [25].

**Table 2:** Small-molecule therapies in CD CD: Crohn’s disease, JAK: Janus kinase, TGF: transforming growth factor, STAT: transducers and activators of transcription, S1P: sphingosine-1-phosphate, DHODH: dihydroorotate dehydrogenase, PDE4: phosphodiesterase-4 Source reference: [24-40]

Drug category	Drug name	Mechanism of action	Use and dosage	Clinical efficacy
JAK inhibitors	Upadacitinib, filgotinib, tofacitinib	Inhibit JAK-STAT signaling → ↓ cytokine activity	Induction and maintenance. Upadacitinib: oral 45 mg daily induction → 15/30 mg maintenance (U-EXCEED, U-EXCEL, U-ENDURE). Filgotinib: oral 200 mg daily (FITZROY, DIVERGENCE-2). Tofacitinib: 10 mg BID induction, 5-10 mg BID maintenance (used in UC; CD trials limited)	Upadacitinib: remission 34-36% wk12 vs. 11-14% placebo (U-EXCEED/EXCEL). U-ENDURE wk52 remission 36-46% vs. 18% placebo. Filgotinib: FITZROY wk10 remission 47% vs. 23% placebo; DIVERGENCE-2 showed fistula healing benefit. Tofacitinib: limited efficacy in CD, mainly used in UC
S1P receptor modulators	Ozanimod, etrasimod	Bind S1P receptors → prevent lymphocyte egress from lymph nodes	Induction and maintenance. Ozanimod: oral 0.92 mg daily (TRUE NORTH, STEPSTONE). Etrasimod: oral once daily (ELEVATE, CULTIVATE)	Ozanimod: STEPSTONE (phase II CD): endoscopic/histologic improvement. Etrasimod: CULTIVATE phase IIb: endoscopic + symptomatic improvement in moderate-to-severe CD
PDE4 inhibitors	Apremilast	Inhibits PDE4 → ↑ cAMP → ↓ pro-inflammatory cytokines	Induction and maintenance. Oral 30 mg BID (LIBERATE, APPRAISE, ongoing CD studies)	Early CD trials suggest modest benefits; not widely used compared to JAK or S1P agents
SMAD7 blockers	Mongersen	Antisense oligonucleotide → restores TGF-β1 anti-inflammatory signaling	Induction. 10-160 mg oral daily (phase II RCT Bovivant et al.; phase III halted)	Phase II: remission 55-65% at day 15 (40/160 mg). Phase III stopped due to no efficacy difference
DHODH inhibitor	Vidofludimus calcium (IMU-838)	Inhibits DHODH, blocking pyrimidine synthesis in activated lymphocytes	Phase II trials ongoing	Promising immunomodulatory effects in early studies; safety comparable to placebo

Janus Kinase Inhibitors

Four intracellular tyrosine kinase proteins are referred to as JAKs: non-receptor tyrosine-protein kinase 2 (TYK2), JAK1, JAK2, and JAK3. JAK3 excludes hematopoietic cells, although the other three proteins are present elsewhere. Signal transducers and activators of transcription (STATs) are cytosolic DNA-binding proteins activated by JAKs in pairs through cytokine binding. The JAK-STAT signaling pathway influences hematopoiesis and innate and adaptive immunity and modulates inflammatory responses while also sustaining the structural and functional integrity of the intestinal epithelium [24,26,27]. In IBDs, JAKs have been demonstrated to be elevated [24,28-30]. For instance, Th2 and Th17 differentiation, as well as B- and T-cell activity, are enhanced by JAK1/JAK3 activation. The antiviral response, wound healing, and B- and T-cell activity are all impacted by JAK1/JAK2/TYK2. Cytopenia may result from the inhibition of JAK2, which is essential in hematopoiesis [24,28]. Notably, IL-13 activates JAK1/JAK3/TYK2, which are implicated in B-cell activation, the epithelial barrier function, and the Th2 anti-inflammatory response. The function of the intestinal barrier is significantly impacted by IL-13 [24,29]. As a result, JAKs are now a therapeutic focus for IBD. Unlike monoclonal antibodies, which typically act on a single target, JAK inhibitors interfere with multiple cytokine signals involved in the inflammatory process [24,30,31]. Multiple oral JAK inhibitors are already in use, each with a unique selectivity and safety profile.

Tofacitinib

As a pan-kinase inhibitor, tofacitinib selectively inhibits JAK1 and JAK3 [32]. Tofacitinib has been developed for CD. In the phase II trial, tofacitinib did not differ significantly from placebo in clinical response during the eight-week induction phase or in maintaining the response at 26 weeks among responders. Clinical response was defined as a decrease of at least 100 points in the Crohn’s Disease Activity Index (CDAI) from baseline, and remission was defined as a CDAI score of 150 or less [33]. Furthermore, an inadequate clinical response and a high risk of infections (50%) throughout the open-label 48-week extension resulted in a high dropout rate (41.3%) among patients in remission at 26 weeks [34]. Thus, tofacitinib's development for CD was halted.

Tofacitinib, a pan-JAK inhibitor with limited selectivity, may explain its side effects and lack of efficacy in CD. More selective JAK inhibitors have since been developed to improve the benefit-to-risk balance.

Upadacitinib

Recently licensed for the treatment of rheumatoid arthritis, upadacitinib (RINVOQ, AbbVie, USA) is a JAK1-selective inhibitor with a half-life of approximately four hours [35]. Upadacitinib's safety and effectiveness in treating individuals with moderate to severe CD were assessed in a double-blind phase II trial [36]. Ninety-six percent of the patients enrolled had previously been treated with one or more anti-TNF-α, which either failed to control their condition or caused adverse effects. At week 16, 13% of patients who received a dosage of 3 mg, 27% of patients who received 6 mg (p < 0.1 compared to a placebo), 11% of patients who received 12 mg, 22% of patients who received 24 mg twice daily, and 14% of patients who received 24 mg of upadacitinib once daily experienced clinical remission, compared to 11% of patients who received a placebo. Except for the 6 mg dosage, which was administered to a small number of patients (n = 37), the effect on clinical remission in CD was not particularly strong. There was no significant dose-response association. Using 24 mg of upadacitinib once or twice daily resulted in endoscopic remission in 14% and 22% of patients, respectively. This benefit was statistically significant but not clinically meaningful. Serious infections occurred in 4% of the treated individuals [36].

In conclusion, taking upadacitinib once daily has the potential to help UC patients experience remission. Further studies are necessary to evaluate the long-term efficacy and safety of upadacitinib, particularly to assess if its selectivity leads to different results compared to tofacitinib. Regarding its use in CD, the evidence from earlier trials remains inconclusive and does not definitively demonstrate a clear benefit.

Filgotinib

Filgotinib is an oral agent that primarily targets JAK1 and has been developed to treat various inflammatory conditions. In a phase II trial by Reinisch et al. that studied the efficacy and safety of filgotinib in perianal fistulizing CD, 47.1% of patients who received filgotinib 200 mg had a combined clinical and MRI-confirmed response at week 24, compared to 29.2% of those receiving filgotinib 100 mg and 26.7% of the patients taking a placebo. Although serious adverse events were more common in the filgotinib 200 mg group than in the placebo group (29.4% vs. 6.7%), there were no treatment-related serious events or deaths [37].

To sum up, JAK inhibitors are a promising family of medications that have a quick onset of action and can be used to induce and maintain remission in UC and CD. Nonetheless, variations in safety and effectiveness have been noted, most likely due partly to variations in JAK selectivity. A long-term evaluation of their safety is recommended due to the significant adverse events, infections, cancers, and cardiovascular and thromboembolic events that have been reported in clinical studies. Before starting treatment, each patient's risks and advantages should be carefully weighed and discussed. Gut-restricted and selective JAK inhibitors have the potential to significantly increase this therapy class's safety in the years to come.

Modulators of Sphingosine 1-Phosphate Receptors

Five distinct subtypes of G-protein-coupled receptors (S1PR1-5) are activated extracellularly by S1P, a lysophospholipid signaling molecule derived from the membrane that acts as a lipid mediator outside the cell [38,39]. Distributions of S1P receptors vary. Subtypes 1, 4, and 5 are essential in immune system control, whereas subtypes 2 and 3 may be associated with unfavorable heart, lung, and cancer events. S1PR1 modifies immunity by preventing lymphocytes from leaving the lymph node and being transported to inflammatory tissues [40]. When the agent is stopped, these effects are undone [39].

Ozanimod was studied in 69 patients with moderate to severe CD in the STEPSTONE phase II uncontrolled prospective observer-blinded endpoint (PROBE) experiment [41]. Over a week, patients received increasing therapy dosages until they reached a dose level of 1 mg of ozanimod once a day. The endoscopic response was 23.2% (95% CI 13.9-34.9), the clinical response was 56.5% (95% CI 44.0-68.4), the clinical remission was 39.1% (95% CI 27.6-51.6), and the endoscopic response was 10.1% (95% CI 4.2-19.8) at week 12. The most often reported significant adverse events were abdominal abscesses (3%) and CD problems (9%). Without a control group, conclusions regarding therapeutic interest cannot be made [41].

Additional small-molecule drugs

Anti-integrins

The goal of anti-integrins is to disrupt the movement of immunological cells, particularly leukocytes. Integrins are heterodimeric glycoproteins that identify and bind cell adhesion molecules (CAMs). They have one α and one β subunit and several structural isoforms. Among these integrins are α4β1 (found in the majority of leukocytes) and α4β7 (found in lymphocytes of the digestive tract). With natalizumab, anti-α4β1 integrins were initially explored as a treatment option [42]. Nevertheless, its application in IBD patients was hindered by the development of progressive multifocal leukoencephalopathy, which can be attributed to the role of α4β1 in mediating lymphocyte trafficking in the brain and stomach [43]. In contrast, vedolizumab and etrolizumab were more effective at inhibiting α4β7 in CD and UC.

The gut-restricted peptide PN-943 (Protagonist Therapeutics Inc., USA) is taken orally and targets MAdCAM1 [44]. Treatment with 1000 mg daily for two weeks revealed PN-943 was well tolerated in a phase I trial in eight patients, with a 94% blood receptor occupancy (manufacturer data). Inclusions are anticipated to be completed in June 2022, and the phase II study is now underway. Additionally, PF-00547659 (ontamalimab), a more sophisticated contender, blocks the connection between MAdCAM1 and the α4β7 integrin [45]. In the 12-week phase II trial (TURANDOT), 357 patients with active UC (total Mayo score ≥6 and endoscopic score ≥2) were enrolled [46]. Among them, 152 patients had not previously been treated with anti-TNF-α therapies, while 205 had prior exposure to these agents. Patients were randomly assigned to receive a placebo or 7.5, 22.5, 75, or 225 mg of PF00547659. At 12 weeks, the three lowest active groups experienced significantly higher rates of remission (total Mayo score ≤2 with no sub-score >1) (11.3%, 16.7%, and 15.5%, respectively) compared to the placebo group (2.7%). Interestingly, anti-TNF-α naive patients exhibited a significantly greater rate of remission in the same active groups compared to those with prior anti-TNF-α exposure (16.7-23.3% vs. 7.3-9.8%). To evaluate the long-term safety of PF-00547659, patients who finished the 12-week course of treatment were then enrolled in the 144-week open-label extension experiment TURANDOT II. On the other hand, PF-00547659 did not show any effectiveness in CD [47].

Inhibitors of Phosphodiesterase-4

The intracellular enzyme PDE4 breaks down cyclic AMP in various cells, including T-cells, immune cells, and macrophages. Activation of nuclear transcription factor kappaB through this catabolic pathway enhances the synthesis of multiple pro-inflammatory cytokines and diminishes the generation of anti-inflammatory factors [48]. To date, no drug candidate has progressed beyond phase II trials [49]. A phase II, double-blind, placebo-controlled RCT has assessed the effects of the PDE4 inhibitor apremilast (Otezla, Celgene®, Summit, NJ, USA), which is authorized for treatment in psoriatic arthritis and Behçet's disease [50]. After 12 weeks, patients treated with 30 mg of apremilast achieved a significantly higher clinical remission rate compared to those receiving placebo (31.6% vs. 12.1%, p = 0.01). Clinical remission was defined as a total Mayo score of 2 with no individual sub-score exceeding 1. Interestingly, the 40 mg dose did not reach statistical significance (21.8% vs. 12.1%, p = 0.27). Moreover, only the 30 mg group demonstrated a significant benefit in mucosal healing at 12 weeks, with 33% of patients achieving healing, compared to 15.5% in the placebo group (p = 0.03). Mucosal healing was defined as a Mayo endoscopic score of 1 and a Geboes score of less than 2 [24]. The medication was generally well tolerated, with only one case of pancreatitis reported in a patient on 40 mg who had a history of hepatobiliary disease. In the 30 mg group, headache and nausea were the most frequent adverse events, occurring in 21.1% and 5.3% of patients, respectively. These rates were slightly higher in the 40 mg group, at 25.5% for headache and 10.9% for nausea [24].

Blockers of SMAD-7

As a therapeutic mechanism, SMAD targeting in IBDs occurs upstream from other medications. Once phosphorylated, the intracellular protein SMAD prevents transforming growth factor-β1 (TGF-β1) from having its anti-inflammatory effects. A major player in this process is SMAD7, and SMAD7 blockers work to restore TGF-β1's anti-inflammatory properties [51,52].

Mongersen (GED-0301; Celgene®) is an antisense oligonucleotide that raises TGF-β1 activity by blocking the transduction of the SMAD7 protein. A phase II randomized, double-blind, placebo-controlled trial (Boirivant et al.) assessed the effects of three different Mongersen doses (10 mg, 40 mg, and 160 mg) in patients with moderate to severe CD [53].

Among the 160 patients analyzed, the two highest doses of mongersen, 40 mg and 160 mg, resulted in significantly higher rates of clinical remission (55% and 65%, respectively) compared to the placebo and 10 mg dose groups, which showed remission rates of 10% and 12%, respectively. Clinical remission was defined as a CDAI score below 150 at day 15, maintained through day 28. For 65% of the patients taking 160 mg daily, a response was obtained by day 15. There were nine serious adverse events (5.6%), most of which were linked to the progression of the illness. However, the subsequent phase III trial was prematurely stopped after 78.6% (551 of 701) of participants completed the study, as Mongersen and placebo demonstrated similar clinical remission rates at 12 weeks (22.8% vs. 25.0%, p = 0.621). As a result, development of Mongersen was discontinued permanently [54].

A novel substance called IMU-838 (Vidofludimus Calcium, Immunic Therapeutics Inc., USA) prevents the human enzyme dihydroorotate dehydrogenase (DHODH) from working [55]. DHODH is highly expressed in proliferating or activated lymphocytes and is essential for synthesizing pyrimidines. By blocking the DHODH, IMU-838 can thereby target activated and quickly growing lymphocytes. This results in greater apoptosis of activated lymphocytes and a reduction in the production of key pro-inflammatory cytokines, such as IL-17A, IL-17F, and IFNγ. In studies conducted on rheumatoid arthritis and multiple sclerosis, vidofludimus calcium demonstrated a placebo-like safety profile [56-58].

Comparing biologic therapy and small-molecule therapy

Efficacy

CD is characterized by mild, moderate, and severe remission; however, there is no standard rule for classifying the disease. Mild may be considered when the disease has minimal impact on the patient's day-to-day activities, eating and drinking normally, <10% weight loss, no features of obstruction, and abdominal mass, and CRP is usually increased above the upper limit of normal. A CDAI of 150-220 is considered mild. Severe disease shows signs of cachexia, obstruction, treatment-resistant symptoms, and increased CRP with a CDAI of >450. Meanwhile, moderately, the two parameters are between [59].

Biologics are highly effective in moderate to severe CD. It has shown promising results in the induction and maintenance of remission. In an RCT by Brian et al., trials of infliximab, adalimumab, and certolizumab pegol were conducted in patients with moderate to severe CD, according to CDAI. Three randomized clinical trials have demonstrated that infliximab is effective in multiple treatment outcomes among patients who responded to induction therapy. Specifically, it increased clinical remission rates (RR 2.50; 95% CI 1.64-3.80), improved clinical response (RR 1.66; 95% CI 1.00-2.76), had a corticosteroid-sparing effect (RR 3.13; 95% CI 1.25-7.81), and helped maintain fistula healing (RR 1.87; 95% CI 1.15-3.04) [60].

In a randomized, double-masked, placebo-controlled, multicenter efficacy and safety study conducted by Colombel et al. Adalimumab 40 mg weekly was administered at 26 weeks, and at 47 weeks, 47% of patients were in remission, compared to 41% at 56 weeks, a significant difference compared to placebo (p = 0.001 vs. placebo) [61]. In Schreiber et al.'s randomized double-blind, placebo-controlled trial, administration of 400 mg of certolizumab pegol via subcutaneous injection at weeks 0, 2, and 4, followed by administration every four weeks, showed better maintenance therapeutic value of 64% compared to placebo at six weeks with continued maintenance of 62% through 26 weeks versus 34% of those receiving placebo (p < 0.001) who had responded to induction treatment with certolizumab pegol [62].

Tofacitinib and upadacitinib are notable JAK inhibitors that have been approved for CD treatment. They block JAK enzymes, which are crucial for inflammatory signaling pathways. Small molecules like JAK inhibitors and S1P receptor modulators are seen as promising treatments for IBD due to their predictable pharmacokinetics, lower likelihood of inducing immune responses, and the convenience of oral administration [63]. In a randomized, double-blind, placebo-controlled phase II study, the FITZROY STUDY conducted by Vermiere et al., 174 people with moderate to severe CD were enrolled (130 in the ilgotinib 200 mg group and 44 in the placebo group). Among them, 47% showed remission at week 10, while only 23% showed remission with placebo (difference of 24 percentage points (95% CI 9-39), p = 0·0077) [64]. Although studies regarding the efficacy of JAK inhibitors as a treatment for CD have been less conclusive, they have certainly captured the researcher's attention due to their high oral bioavailability [65].

Route of Administration

The main challenge while prescribing medications for any disease is to ensure the correct dosage that sufficiently targets the diseased area and has minimal adverse effects. To achieve a significant result, the route of administration is of immense value, as it determines bioavailability, systemic absorption, the desired action on the disease, and potential adverse effects [66]. Different drug delivery systems are being researched to achieve therapeutic efficacy with minimal adverse effects [67].

Biologics that have shown promising results in treating CD are administered either subcutaneously or intravenously. The meta-analysis conducted by Elford et al. comparing the efficacy of intravenous and subcutaneous biologics demonstrated no significant difference between the two. Both have shown similar efficacy in terms of the route of administration [68].

The IBD treatment landscape has significantly broadened over the last decade. New oral therapies, particularly small-molecule drugs, are emerging as viable alternatives to traditional biomolecular therapies. This shift is crucial for improving patient outcomes in IBD management. S1P receptor modulators represent the latest class of oral small molecules approved by the FDA for the treatment of UC. These modulators are currently under investigation for their effectiveness in CD, another form of IBD [69]. Similarly, a clinical trial by Monteleone et al. in which oral SMAD7 antisense oligonucleotide was administered at 10, 40, and 160 mg versus placebo per day for two weeks. Clinical remission was achieved on day 15, showing a positive result in managing CD. At day 15, 55% of individuals receiving the 40 mg dose achieved the primary treatment goal of remission, characterized by a CDAI score of less than 150.

In comparison, 65% achieved the primary endpoint of a CDAI score of 160, compared with 10% on placebo (p = 0.001) [54]. Small molecular therapies, such as JAK inhibitors like upadacitinib and tofacitinib, are also administered orally. However, there are many challenges in oral form, such as drug interaction with colon microorganisms, the pH of the colon environment, and disease processes that alter the cellular permeability. To keep these barriers in check, oral drug forms are being processed using nanoparticles and microparticles [70].

Onset of Action

With infliximab, clinical improvement can be seen as early as two to four weeks, with a significant response of 8-12 weeks. In the ACCENT I trial, 573 patients with CD and a CDAI score of at least 220 were enrolled. Following a single infusion of infliximab, 58% (335 patients) showed a clinical response within two weeks. At week 30, remission was achieved in 21% of patients in group I (23 out of 110), compared to 39% in group II (44 out of 113; p = 0.003) and 45% in group III (50 out of 112; p = 0.0002) [71]. Similarly, while researching vedolizumab, there was a slower onset with noticeable improvement by 6-10 weeks and maximal effect by 14 weeks. In the GEMINI II trial, vedolizumab demonstrated clinical remission in 14.5% (n = 186) of patients versus 7.6% (n = 131) with placebo in the induction phase till week 6 and 39% (n = 143) versus 22.6% (n = 146) with placebo in the maintenance phase till week 52 [72]. For ustekinumab, clinical response can be seen as early as three to six weeks, with sustained improvement over time. In the UNITI-1 trial, conducted on 741 patients who met the criteria for primary or secondary nonresponse to TNF antagonists or had unacceptable side effects, ustekinumab at a 130 mg single intravenous dose showed a clinical response in 34% of patients by week 6 versus 21.5% receiving placebo (p < 0.003) [73].

In the STEPSTONE trial, 69 people with moderate to severe CD were enrolled. Ozanimod demonstrated a clinical response in 56.5% of patients by week 12 and achieved clinical remission (CDAI <150 points) in 39.1% [41]. Biologics (e.g., infliximab) tend to have a moderate onset of action, with anti-TNF agents showing the fastest response in two to four weeks. Small-molecule therapies (e.g., tofacitinib, ozanimod) can act quickly, with some patients experiencing improvement within one to four weeks. The choice depends on the patient's clinical profile, disease severity, and treatment goals.

Dosing and Frequency

Managing CD with biologics involves careful consideration of dosing and frequency to optimize therapeutic outcomes. Biologics, including anti-TNF agents and newer therapies, can be administered at standard or escalated doses depending on patient response and disease severity. Biologics, such as infliximab and adalimumab, are typically initiated with a loading dose, followed by maintenance doses. For instance, infliximab is administered at weeks 0, 2, and 6, then every eight weeks [74]. Standard maintenance dosing has shown effectiveness in achieving mucosal healing, with anti-TNF agents demonstrating significant efficacy compared to placebo [75]. Approximately one-third of patients may experience loss of response, necessitating dose escalation or increased frequency [76]. In contrast, while dose escalation can improve outcomes for some patients, it may also increase the risk of adverse events, particularly with anti-TNF agents [74]. Therefore, a balanced approach that considers efficacy and safety is essential in managing CD with biologics [31].

Managing CD with small molecules involves various dosing strategies and frequencies, reflecting the complexity of treatment needs. Recent studies highlight the efficacy and safety of small-molecule drugs, including JAK inhibitors and S1P modulators, which have shown promise in clinical trials. Tofacitinib has effectively achieved primary endpoints in clinical trials, with dosing typically starting at 10 mg twice daily, followed by a maintenance dose of 5 mg twice daily [74]. In a trial, Semapimod was administered intravenously at 60 mg for one to three days, with cumulative dosing showing some efficacy in a limited patient subset [77]. While small molecules offer new avenues for managing CD, the variability in patient response necessitates personalized treatment plans. Conversely, the potential for adverse effects and the need for ongoing monitoring remain critical considerations in therapy management [78].

Cost of Treatment

Due to the positive results of biologics and small-molecule therapies in managing CD, many physicians are leaning towards these approaches to manage the condition. These medicines have uplifted the lives of many affected individuals. Though it has been an outstanding treatment option, we cannot ignore the financial burden it has brought with it. These therapies can be expensive, so analyzing their associated costs is crucial for healthcare planning and resource allocation. In a study conducted on German patients with IBD, the mean IBD-related direct healthcare cost per patient-year for bio-naive CD patients treated with anti-TNF-α was €30,246, while for vedolizumab it was €28,227. For bio-experienced patients, costs were €34,136 and €32,112, respectively [79]. A study found no significant cost difference between infliximab (£6,877) and adalimumab (£8,226) over one year, suggesting that choice may depend more on patient-specific factors than on cost alone [80]. The median annual cost of treatment for biologics is $92,000 (IQR 31,000-357,000), while for small-molecule drugs, it is $33,000 (IQR 4,000-177,000), indicating significantly higher costs associated with biologics in the management of CD [81]. The combination of lower development costs, shorter market exclusivity periods, fewer patents, and lower annual treatment costs contributes to the overall lower costs associated with small-molecule therapies compared to biologics [81].

Combination therapy versus monotherapy

Combination therapy demonstrates significant advantages over monotherapy in the management of CD, particularly in cases of moderate to severe disease or refractory IBD. Evidence from cohort studies and case series involving more than ten patients has underscored the safety and efficacy of combining biologic agents or biologics with small molecules such as tofacitinib. Tofacitinib, currently approved for both induction and maintenance therapy in moderate to severe UC, has shown enhanced effectiveness when utilized alongside biologicals in refractory cases, potentially overcoming the "ceiling effect" of monotherapy [82].

Dual-targeted therapies, which engage distinct inflammatory pathways, such as vedolizumab or ustekinumab combined with another biologic or small molecule, such as tofacitinib (an oral small molecule preferential JAK1 and JAK3 inhibitor), represent an optimal treatment approach, particularly for patients with refractory disease or extra-intestinal manifestations, due to their effect on different inflammatory pathways [82]. The greater safety profiles of newer biologics, such as vedolizumab and ustekinumab, further reinforce their role as anchor therapies in combination regimens, offering a promising solution for refractory cases [82].

Drug-drug combination therapy has emerged as a promising approach in managing CD and other forms of IBD, particularly in patients with refractory disease or those with extra-intestinal manifestations [83]. Current evidence supports that combining infliximab and thiopurines is more effective than monotherapy in inducing and maintaining remission. The SONIC RCT showed that clinical remission with infliximab (44.4%) and azathioprine (30.0%) monotherapy was less than the combination of both (56.8%). Mucosal healing at week 26 for CD patients was 30.1%, compared to 16.5% for infliximab and 43.9% for combination therapy [84]. Combining infliximab with immunosuppressors such as thiopurines or methotrexate has enhanced infliximab pharmacokinetics and improved outcomes. However, this combination also carries an increased risk of infections and malignancies.

DBT has demonstrated efficacy in various studies, notably a 2018 case series involving 10 patients (four with CD, six with UC) treated with anti-TNF agents and vedolizumab, who reported clinical remission in all patients after a follow-up of 10 to 17 months [85]. Similarly, a retrospective study involving 15 patients with IBD undergoing DTT observed clinical response in 73% of patients, a 67% reduction in steroid use, and endoscopic improvement in 44% after six months [82]. A Canadian cohort study of 22 patients with CD reported clinical remission in 41% and endoscopic improvement in 43% after a median duration of nine months [19]. These findings highlight the potential of drug-drug combination therapy to improve clinical and endoscopic outcomes, particularly for patients who have failed conventional treatment strategies.

Despite the advances in biologic therapies for CD, surgery remains crucial for many patients. While biologics have undoubtedly improved disease control for many, a substantial number of patients do not benefit fully from these treatments and require major abdominopelvic surgery after exposure to systemic and gut-selective biologics with worsened disease severity [86].

Steroid use in CD patients remains a challenge, with 30-40% of patients treated for moderate to severe CD, resulting in steroid dependency and inability to taper off steroids by the time of surgery. Vedolizumab, a gut-selective drug, presents a lack of systemic effects; as such, it received great enthusiasm following the FDA's approval for its safety profile. Thus far, numerous studies highlight that steroids and vedolizumab both present similar postoperative complications that manifest as surgical site infections and intra-abdominal sepsis when exposed preoperatively [87].

Contrarily, other studies report no increased risk, making the impact of biologics on postoperative outcomes still a topic of ongoing research. Thus, although biologics have improved disease control, they have not eliminated the need for surgery in 60-80% of CD patients [88].

Long-term treatment of Crohn's disease

Long-term treatment of CD significantly impacts compliance, lifestyle, economic burden, and disease recurrence. Managing long-term treatment regimens for patients with CD can be difficult because of it being a chronic disease and the frequent medication schedules, as well as the potential side effects to consider. For instance, a young patient might present with abdominal pain, chronic diarrhea, weight loss, and fatigue [89]. Evaluating the severity of their condition and prognostic factors is vital for guiding effective treatment. Additionally, other forms of IBD, like UC, can also experience seasonal flare-ups [90], highlighting the need for robust management strategies. Non-compliance with treatment can increase the risk of flare-ups and complications associated with CD.

Treatment adherence is crucial for remission of CD. The treatment plan includes medication regimens, lifestyle modifications, and regular follow-up appointments as recommended by the healthcare provider. Consistent adherence helps maintain remission and reduces the risk of complications [91].

Corticosteroids and TNF inhibitors are critical management and highly effective medications for inducing remission. To maintain remission, options include 5-aminosalicylic acid products, immunomodulators such as azathioprine, 6-mercaptopurine, and methotrexate, as well as TNF inhibitors like infliximab, adalimumab, certolizumab, and golimumab [92].

Patients with CD may encounter challenges with adherence to treatment due to factors such as medication side effects, the complexity of treatment regimens, psychological stress, financial limitations, or insufficient understanding of the disease. Non-adherence to treatment can result in a higher risk of disease flares, complications, hospitalizations, and reduced quality of life [93].

Adherence to treatment plays a crucial role in managing CD and improving patient outcomes. Studies demonstrate that compliance with biologic therapies can significantly reduce relapse rates and enhance long-term outcomes [94]. Conversely, poor adherence to maintenance medications, such as 5-aminosalicylates or immunosuppressants, has been associated with increased rates of disease recurrence [95]. Addressing barriers to adherence through education, simplified regimens, and support systems can significantly enhance treatment compliance and improve outcomes in CD.

Improving dietary and medication adherence involves a multifaceted approach. Patient education is fundamental, as understanding the importance of consistent medication use and proper dietary choices can help reduce symptoms and prevent complications. Collaboration with dietitians to develop personalized, flexible meal plans allows patients to identify and avoid trigger foods while ensuring nutritional adequacy. Simplified medication regimens, such as once-daily dosing or combination therapies, can further promote adherence. Additionally, practical tools like pill organizers and medication reminder apps have proven effective in minimizing missed doses [96]. Counseling on proper medication administration, particularly for biologics, also supports improved compliance [93,94].

Psychological and emotional support is equally critical in fostering adherence. Mental health challenges such as stress, anxiety, or depression can negatively impact a patient's ability to adhere to treatment plans, underscoring the importance of counselling and therapy. Peer support groups provide opportunities for shared experiences and motivation, helping patients stay committed to their care plans. Regular follow-ups and shared decision-making between patients and providers ensure that treatments are tailored to individual needs, enhancing adherence [96].

Economic barriers are another significant challenge. Financial assistance programs and accessible resources can alleviate the economic burden associated with treatment, making it easier for patients to comply [97]. By addressing these diverse challenges, healthcare providers can foster better adherence to treatment regimens and improve clinical outcomes in patients with CD.

## Conclusions

CD remains a lifelong, relapsing condition with no definitive cure. Current management strategies focus on symptom control and inflammation suppression. However, emerging research in precision medicine, gut microbiome modulation, and novel biologic therapies is allowing for a more personalized and potentially curative approach. Biologics have been the mainstay for moderate to severe CD, particularly in steroid-dependent or refractory cases, while small molecules provide oral alternatives with potentially fewer systemic effects. Dietary interventions have also gained prominence, with CDED emerging as a promising whole-food approach to reducing exposure to pro-inflammatory dietary components and managing symptoms. Additionally, stem cell therapy is gaining traction as a novel treatment for CD-associated fistulas, particularly with the use of mesenchymal and adipose-derived stem cells; however, further research is needed to assess its efficacy in intestinal involvement.
